# Effectiveness of blood flow restricted exercise compared with standard exercise in patients with recurrent low back pain: study protocol for a randomized controlled trial

**DOI:** 10.1186/s13063-016-1214-7

**Published:** 2016-02-12

**Authors:** Shinichi Amano, Arimi Fitri Mat Ludin, Rachel Clift, Masato Nakazawa, Timothy D. Law, Laura J. Rush, Todd M. Manini, James S. Thomas, David W. Russ, Brian C. Clark

**Affiliations:** Ohio Musculoskeletal and Neurological Institute (OMNI), Ohio University, 250 Irvine Hall, 1 Ohio University, Athens, OH 43147 USA; Clinical and Translational Research Unit (CTRU), Ohio University, Athens, OH 45701 USA; Faculty of Health Sciences, Universiti Kebangsaan Malaysia, Kuala Lumpur, Malaysia; Office of Research and Grants, Ohio University Heritage College of Osteopathic Medicine, Athens, OH 45701 USA; Department of Biomedical Sciences, Ohio University, Athens, OH 45701 USA; Department of Family Medicine, Ohio University, Athens, OH 45701 USA; Department of Geriatric Medicine, Ohio University, Institute on Aging, Athens, OH USA; Department of Geriatric Medicine, University of Florida, 2004 Mowry Road, PO Box 100107, Gainesville, FL 32611 USA; Division of Physical Therapy, The School of Rehabilitation and Communication Sciences, Ohio University, Athens, OH 45701 USA

**Keywords:** Low back pain, Blood flow restriction, KAATSU, Trunk extensor, Muscle, Strength, Endurance, Exercise

## Abstract

**Background:**

Low back pain is a highly prevalent condition in the United States and has a staggeringly negative impact on society in terms of expenses and disability. It has previously been suggested that rehabilitation strategies for persons with recurrent low back pain should be directed to the medial back muscles as these muscles provide functional support of the lumbar region. However, many individuals with low back pain cannot safely and effectively induce trunk muscle adaptation using traditional high-load resistance exercise, and no viable low-load protocols to induce trunk extensor muscle adaptation exist. Herein, we present the study protocol for a randomized controlled trial that will investigate the “cross-transfer” of effects of a novel exercise modality, blood flow restricted exercise, on cross-sectional area (primary outcome), strength and endurance (secondary outcomes) of trunk extensor muscles, as well as the pain, disability, and rate of recurrence of low back pain (tertiary outcomes).

**Methods and study design:**

This is a single-blinded, single-site, randomized controlled trial. A minimum of 32 (and up to 40) subjects aged 18 to 50 years with recurrent low back pain and poor trunk extensor muscle endurance will be recruited, enrolled and randomized. After completion of baseline assessments, participants will be randomized in a 1:1 ratio to receive a 10-week resistance exercise training program with blood flow restriction (BFR exercise group) or without blood flow restriction (control exercise group). Repeat assessments will be taken immediately post intervention and at 12 weeks after the completion of the exercise program. Furthermore, once every 4 weeks during a 36-week follow-up period, participants will be asked to rate their perceived disability and back pain over the past 14 days.

**Discussion:**

This study will examine the potential for blood flow restricted exercise applied to appendicular muscles to result in a “cross-transfer” of therapeutic effect to the lumbar musculature in individuals with low back pain. The results of this study will provide important insights into the effectiveness of this novel exercise modality, which could potentially provide the foundation for a cost-effective and easy-to-implement rehabilitation strategy to induce muscle adaptation in the absence of high mechanical and compressive loading on the spine.

**Trial registration:**

This trial is registered with ClinicalTrials.gov (registration number: NCT02308189, date of registration: 2 December 2014).

## Background

Low back pain (LBP) is a highly prevalent condition in the United States and has a staggeringly negative impact on society in terms of expenses and disability. It is the most common reason for seeking medical care, resulting in more than 52.3 million physician visits annually in 2012 [1]. Ninety percent of adults will experience LBP in their lifetime, and the recurrence of LBP in the year following an acute episode ranges from 24 to 87 % [2]. Acute occurrence of LBP results in rapid onset of atrophy of the lumbar multifidus muscle [3], and patients with both recurrent and chronic LBP exhibit wasting of the trunk extensors (TEs) [4–9]. The TE muscles of patients with recurrent and chronic LBP are also substantially weaker [10–14] and more fatigable [11, 15–20] than those of healthy individuals. Further, TE weakness is predictive of LBP recurrence [21, 22], and poor endurance is predictive of an increased risk for a first-time episode of LBP and recurrence of LBP [21, 23]. Collectively, these findings indicate that persons with LBP, particularly recurrent LBP, exhibit deconditioned TE muscles [24]. Thus, the enhancement of an individual’s level of muscular fitness using exercise interventions has long been an important goal in the rehabilitation of LBP, and it has previously been suggested that rehabilitation strategies for persons with recurrent LBP should be directed to the medial back muscles as these muscles provide functional support of the lumbar region [25].

Indeed, rehabilitation strategies for LBP often attempt to tackle maladaptive changes in TE muscle morphology and function, and they have been shown to be helpful in the management of LBP [26]. However, according to the most recent Cochrane review, exercise therapy is only “somewhat effective” at decreasing LBP and improving function [27]. Exercise therapy encompasses a variety of interventions varying in type, intensity, and frequency. The most common types of LBP exercises are (1) strengthening, (2) stretching, (3) aerobic, (4) coordination, and (5) mobilizing exercises [28, 29]. Among these, strengthening exercises were the most effective for improving function and ranked third for improving LBP [28]. Numerous studies have shown that low- and moderate-load trunk extension resistance exercise increases TE muscle size, strength, and endurance and also reduces pain and disability in recurrent and chronic LBP [30–35]. However, the overall effectiveness of current exercise therapy protocols would be improved if a greater stimulus for adaptation could be provided to the musculature. While the American College of Sports Medicine recommends high-load exercise for inducing muscle adaptation [36], the vast majority of TE exercises for the rehabilitation of LBP [37] are performed at low loads. This assertion is supported by observations that (1) moderate-load trunk extension exercise reduces disability more than low-load exercise, and (2) high-load trunk extension exercise increases erector spinae muscle volume by 2.2 %, whereas low-load exercises do not induce hypertrophy [38]. With this stated, the reasoning for avoiding high-load exercise is rational due to concern over the high mechanical and compressive loading on the spine [37, 39]. Hence, the problem facing clinicians is that many individuals with LBP cannot safely and effectively induce trunk muscle adaptation using traditional high-load resistance exercise, and no viable low-load protocols to induce TE muscle adaptation exist. Taken together, it is crucial to develop therapeutic approaches utilizing low mechanical loads that can still deliver sufficient stimuli to the TE muscles and reverse the changes in TE muscles commonly observed in patients with LBP.

One potential approach to addressing this paradoxical problem is to utilize blood flow restricted (BFR) exercise (also referred to as KAATSU exercise). This involves performing exercise with low loads while blood flow to the working muscles is partially occluded by a pressure cuff. Evidence collected over the past decade suggests that performing low-load exercise with modest BFR to the exercising muscles serves as a potent stimulus for increasing muscle mass [40–47], strength [40–45, 47–54] and endurance [41, 44, 55, 56]. The mechanisms of BFR exercise have begun to be explored. We recently reported the notion that low-load BFR exercise down-regulates proteolytic gene expression [57], and others have observed that it enhances mammalian target of rapamycin signaling and stimulates muscle protein synthesis [58–60]. Additionally, resistance training with BFR amplifies high-energy phosphate depletion, decreases muscle pH, and maintains this altered metabolic milieu [61]. The subsequent strong stimulation of the metaboreflex may explain the findings from numerous studies, including our own, indicating that a single bout of BFR exercise increases serum growth hormone levels [44, 62–64] comparable to, or greater than, that observed during resistance training at much higher intensities [65].

Without question, BFR exercise cannot be directly applied to muscles of the trunk as it is not feasible to occlude circulation of the thorax. However, there is evidence that BFR exercise not only has hypertrophy-promoting effects on the exercising muscles that are undergoing BFR, but also results in systemic hypertrophy-promoting effects that extend to muscles not exercising under BFR conditions. Madarame et al. reported that 10 weeks of low-load leg extension resistance exercise, with BFR performed prior to low-load elbow flexor exercise without BFR, increased muscle strength (9.8 %) and size (12 %) of the elbow flexor muscle group even though the elbow flexors were trained without BFR [66]. While their findings are promising, this “cross-transfer” of effect of BFR exercise has been investigated minimally and thus further research is needed. In particular, determining whether or not blood flow restricted exercise exerts a “cross-transfer” of effect to muscle groups that stabilize the spine is of interest due to their clinical relevance to LBP. Herein, we present the study protocol for a randomized controlled trial that will generate effect sizes on the effects of BFR exercise on TE cross-sectional area (CSA) (primary outcome), strength and endurance (secondary outcomes), as well as the pain, disability, and rate of recurrence of LBP (tertiary outcomes). We hypothesize that BFR exercise, with the appendicular muscles performed prior to low-load TE exercise, will produce systemic effects that enhance TE muscle adaptation in a dose-response manner beyond those seen with only low-load TE exercise. This study is registered on ClinicalTrials.gov as “The Back Exercises to Neutralize Disability (BEND) Pilot Study” (NCT02308189).

## Methods and study design

**Fig. 1:**
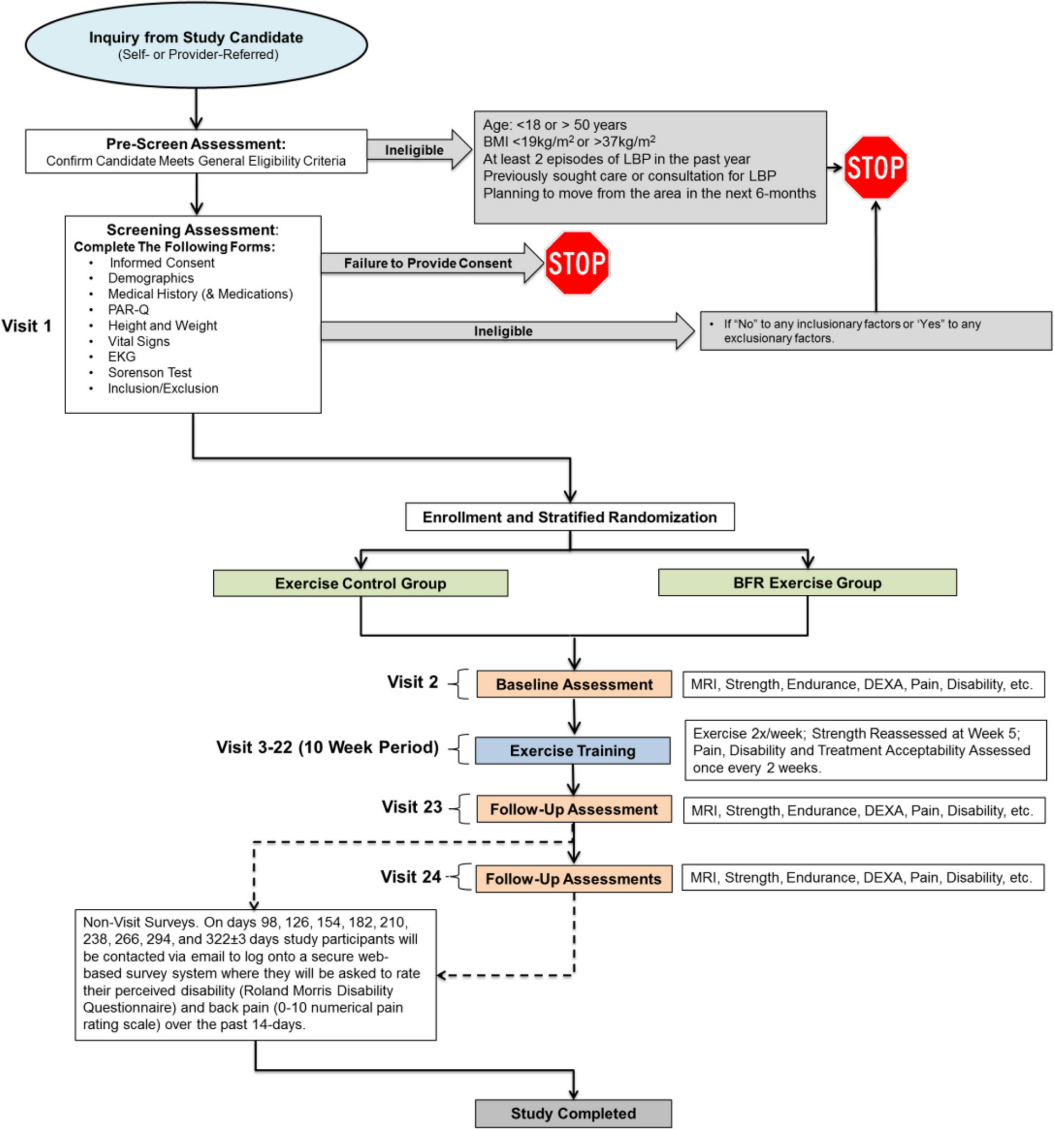
Study design

This is a single-blinded, single-site, randomized controlled trial. It is a two (group) by three (time) repeated measures factorial design. The overall study design is illustrated in Fig. 1.

### Study participants

A minimum of 32 (and up to 40) subjects aged 18 to 50 years with recurrent LBP and poor TE muscle endurance will be recruited, enrolled and randomized in this study (*n* = 16–20/group). Potential participants will be referred by community physicians, other local health care providers, or self-referred. All individuals interested in the study will complete a pre-screening survey via a secure web site. Individuals not ruled ineligible on the basic pre-screen survey will be invited for an in-person screening. Written informed consent will be obtained from the participant. Ethical approval was obtained for this study from the Ohio University’s Institutional Review Board (study number: 14 F025).

Eligible participants will have recurrent LBP. We operationally define recurrent LBP as individuals who answer “yes” to the following question: Have you had two or more episodes of LBP in the past 12 months with at least one of the episodes causing a restriction of work or leisure time activity? They must also exhibit low to moderate trunk extensor endurance (longer than 176 seconds on a modified Sorensen test) to be included in this study [21]. Individuals who have participated in any progressive resistance exercise within the previous 24 weeks prior to screening will be excluded. Table 1 describes the inclusion and exclusion criteria in detail. These criterion are designed to recruit a population with recurrent LBP (e.g., inclusion item 2), but to exclude potential participants who are currently experiencing a level of LBP above where we have concerns about the pain interfering with a participant’s ability to appropriately perform the exercise prescription (e.g., exclusion criteria 4 results in the exclusion of potential participants whose current pain level is 5 or higher on a 0–10 scale).

**Table 1:** Inclusion and exclusion criteria

Inclusion criteria
1. Between 18 and 50 years of age 2. *Answer “yes” to the following questions:* ▪ Have you had two or more episodes of low back pain (LBP) in the past 12 months with at least one of the episodes causing a restriction of work or leisure time activity? 3. Body mass index between 17 and 37 kg/m^2^ 4. With no condition that could limit participation in supervised resistance training exercise based on the Physical Activity Readiness Questionnaire (PAR-Q) 5. Sedentary lifestyle as measured by a classification of “low” or “moderate” levels of physical activity based on scoring criteria of the International Physical Activity Questionnaire (IPAQ) 6. Exhibit low trunk extensor endurance defined as a time to task failure during the modified-Sorensen test of <176 seconds.
Exclusion criteria
1. Participate in progressive resistance exercise within the previous 24 weeks prior to screening 2. Experienced limb amputation (except for toes) and/or any fracture within 24 weeks 3. Have a personal history of the following neurological disorders: Alzheimer’s disease, amyotrophic lateral sclerosis, multiple sclerosis, Parkinson’s disease, or stroke 4. Have a personal history of the following cardiorespiratory disorders: congestive heart failure, myocardial infarction, or peripheral vascular disease 5. Have conditions (e.g., myasthenia gravis, myositis, muscular dystrophy or myopathy, including drug-induced myopathy) leading to muscle loss, muscle weakness, muscle cramps or myalgia 6. Have a personal history of the following musculoskeletal disorders: rheumatoid arthritis, spinal or pathological fractures, scoliosis, spondylolisthesis, avascular necrosis or osteonecrosis, severe osteoarthritis. (Including a history of spinal surgery or a hip arthroplasty). 7. Have a personal history of any of the following conditions or disorders not previously listed: diabetes, fibromyalgia, active cancer, severe obesity (i.e., body mass index greater than 35 kg/m^2^), clinical depression (i.e., subjects who score 24 or higher on the Center for Epidemiology Depression Scale) 8. Have chronic or relapsing/remitting gastrointestinal disorders such as inflammatory bowel diseases, irritable bowel syndrome or gastrointestinal infections within 28 days of screening 9. Have an acute viral or bacterial upper or lower respiratory infection at screening 10. Have moderate or severe chronic obstructive pulmonary disease 11. Report back pain greater than 4 (on a 10- point numerical pain rating scale) at screening 12. Have a leg length discrepancy >3 cm 13. Exhibit abnormal or uncontrolled blood pressure (BP) at the screening visit (i.e., diastolic BP >100 and/or systolic BP >160 mmHg). If taking anti-hypertensive medication, have to be on stable doses of medication for more than 3 months 14. Exhibit abnormal electrocardiogram findings indicative of left ventricular hypertrophy (based on Cornell voltage criteria) at the screening visit 15. Have a current or recent history (within 1 year of screen) of heavy alcohol consumption (men ≥21 drinks/week, 4 drinks/day; women ≥14 drinks/week, 3 drinks/day) or drug abuse 16. Have a current or previous use of any drugs known to influence muscle mass or performance within 24 weeks 17. Report being pregnant, lactating, or that they anticipate becoming pregnant in the next year 18. Report unexplained weight loss over the past month (>10 lbs) 19. Report they have pending litigation related to an episode of LBP or are receiving any type of disability services 20. Use of systemic glucocorticoids within 12 weeks prior to screening 21. Report having received any treatment for LBP by a health care practitioner in the past 6 weeks22. Have contraindications for exposure to a magnetic field

### Randomization and blinding

Participants will be randomized in a 1:1 ratio to receive a 10-week resistance exercise training program with BFR (BFR exercise group) or without BFR (control exercise group). Randomization will be stratified by sex. Because the sample size in each arm is small, permuted-block randomization will be used to ensure equal sample size. Specifically, we will create blocks with varying sizes (i.e., 4–6) within each sex and will permute treatments within each block. Study personnel conducting outcome assessments will remain blinded to group assignment throughout the study. The allocation code and assignment table were generated by a biostatistician (MN). The study participants were enrolled and assigned to the respective interventions by an unblinded project manager (RC).

### Study time line

A table of events for study is illustrated in Table 2. This study will have a screening/baseline assessment period of 21 days, a 10-week exercise training period and a 36-week follow-up period after the last exercise session. Participants will visit Ohio University’s Clinical and Translational Research Unit facilities prior to the intervention for their baseline assessments (detailed below). Following completion of the 10-week exercise intervention, participants will be re-assessed (primary endpoint). Next, participants will enter a 36-week follow-up period during which all outcomes will be assessed 12 weeks (±7 days) after the completion of the exercise program and once every 4 weeks during the follow-up period, they will be contacted via email to log onto a secure web-based survey system where they will be asked to rate their perceived disability (Roland Morris Disability Questionnaire [67]) and back pain (0–10 numerical pain rating scale) over the past 14 days. These data will be used to calculate the rate, duration, pain intensity, and disability of LBP recurrence during the follow-up period.

**Table 2:** Schedule of events for all groups

Study procedure	Screen/baseline period		Intervention period					Follow-up		
	Screen visit 1	Baseline visit 2	Visits 3–6^b^	Visits 7–10^b^	Visits 11–14^b^	Visits 15–18^b^	Visits 19–22^b^	Visit 23^a^	Visit 24^a^	Non-visit surveys
Day (window, ± days)	−21 to −1	−21 to −1	1 − 14	15 − 28	29 − 42	43 − 56	57 − 70	71 ± 4	154 ± 7	Once every 4 weeks^c^
Screening/baseline:										
Informed consent	X									
Inclusion/exclusion	X									
Medical history	X									
Weight	X							X	X	
Height and demographics	X									
PAR-Q, IPAQ	X									
Electrocardiogram	X									
Sorensen test	X									
Randomization			X							
Exercise sessions:										
Exercise^d^			X	X	X^d^	X	X			
Efficacy:										
MRI		X						X	X	
DEXA		X						X	X	
Strength		X						X	X	
Endurance		X						X	X	
Pain and disability	X	X	X	X	X	X	X	X	X	X
Treatment acceptability			X	X	X	X	X			

^a^Efficacy outcome measures may be collected in more than one visit if needed and/or preferred. ^b^Exercise training sessions will be performed twice per week with at least 1 day between the exercise sessions. Once every 2 weeks, prior to performing the exercise, the pain, disability, and treatment acceptability outcomes will be assessed (i.e., pain, disability, and treatment acceptability will be quantified on visits 6, 10, 14, 18, and 22). ^c^On days 98, 126, 154, 182, 210, 238, 266, 294, and 322 ± 3 days study participants will be contacted via email to log onto a secure web-based survey system where they will be asked to rate their perceived disability (Roland Morris Disability Questionnaire) and back pain (0 − 10 numerical pain rating scale) over the past 7 days. ^d^One of the exercise training sessions will be replaced by the strength assessment sessions during week 5.

*DEXA* dual-energy X-ray absorptiometry, *IPAQ* International Physical Activity Questionnaire, *MRI* magnetic resonance imaging, *PAR-Q* Physical Activity Readiness Questionnaire

### Outcome measures

Outcome measures to be assessed at baseline, after the completion of the exercise intervention, and at week 12 of the follow-up period include:

- *Trunk extensor muscle cross-sectional area (primary outcome):* magnetic resonance imaging (MRI) will be performed with a 0.25-Tesla Musculoskeletal MRI system (Esaote G-Scan Brio, Genoa, Italy) to acquire contiguous transverse T-1 weighted spin echo image slices in the trunk region between L2 and L5, with a slice thickness of 10 mm. To ensure the consistency within/among subjects, the isocenter will be positioned at the midpoint of the L3/L4 intervertebral disc. The number of the slices will vary across participants to cover the region of our interest. Prior to all MRIs subjects will lie supine for at least 15 minutes to minimize the effects of fluid shifts on volumetric calculations. The post-testing MRI scan will be obtained 3 days after the final training session to minimize the effects of exercise-induced fluid shifts. After scanning, images will be transferred to a computer for calculation of CSA. Muscle anatomical CSA will be calculated for (1) the quadratus lumborum, (2) iliocostalis lumborum/longissimus thoracis (these two muscles will be grouped due to the difficulty in defining distinct fascial borders in some subjects), and (3) the multifidus. This calculation will be based on an average of three slices obtained from the center of the respective muscles
- *Trunk extensor muscle strength (secondary outcome):* participants will perform three maximum isometric TE contractions on a customized lumbar extension dynamometer (MedX; Ocala, FL, USA) at 36° from full extension (additional trials will be provided as needed if subjects continually exert more force with each trial). Each contraction will last approximately 5 seconds with at least a 60-second interval. Muscle strength will be defined as the highest value recorded in any trial. Muscle strength for leg extensors, plantar flexors, and elbow flexors will also be assessed to permit the calculation of the training intensity for the exercise programs. For all strength assessments, time-series torque signals will be collected at 500 Hz by a Biopac MP150 system (Biopac Systems Inc., Santa Barbara, CA, USA)
- *Trunk extensor muscle endurance (secondary outcome):* participants will perform a sustained, submaximal isometric trunk extension contraction at 20 % of their baseline muscle strength until volitional task failure on a customized lumbar extension dynamometer at 36° from full extension. During this task, the target will be displayed on a computer monitor placed in front of the participant and the time to task failure will be quantified. The time-series torque signal will be collected at 500 Hz by a Biopac MP150 system (Biopac Systems Inc., Santa Barbara, CA, USA)
- *Pain and perceived disability (tertiary outcomes):* a 0–10 numerical pain rating (NPR) scale will be used to quantify LBP. The Roland Morris Disability Questionnaire, which consists of 24 items related to LBP, will be used to quantify perceived disability [67]. These data will be used to calculate the rate, duration, pain intensity, and disability of LBP recurrence during the follow-up period. To analyze LBP recurrence, we will first define an episode of LBP as a period of pain in the lower back lasting for more than 24 hours, preceded and followed by a period of at least 1 month without LBP. This definition is consistent with that suggested by de Vet and colleagues [68]. We will then compute the rate of LBP recurrence as the number of episodes per quarter, duration as how long on average each episode lasted within each participant, and intensity as the mean and maximum pain/disability scores over the follow-up period as well as during an episode of LBP occurrence

Other outcomes:

- *Body composition.* Dual-energy X-ray absorptiometry (DEXA: Hologic Discovery QDR model Series, Waltham, MA, USA) will be performed to assess whole body and regional lean mass as well as whole body and regional fat mass. Participants will be instructed to adequately hydrate prior to the body composition assessment. These data will permit us to determine the effects of the exercise interventions on increasing appendicular lean mass and examine whether individuals with the greatest increases in appendicular lean mass exhibited the greatest “cross-transfer” of effect to the TE CSA (measured via MRI)
- *Treatment acceptability*. Treatment acceptability will be determined by administering the Treatment Evaluation Inventory survey at the end of every fourth exercise session [69]

### Exercise interventions

Supervised exercise training sessions will be conducted twice per week for 10 weeks. For both the BFR exercise group and the control exercise group the exercise intensity will be performed at 25 % of maximal isometric strength. The rationale for basing the exercise intensities on maximal isometric force (as opposed to 1 repetition maximum) is to minimize the potential for injury associated with performing a maximal dynamic trunk extension task. Strength will be re-assessed at the mid-point of the exercise training (i.e., 5 weeks) and exercise intensity values adjusted accordingly. Participants in the BFR exercise group will perform three sets of leg extension, plantar flexion and elbow flexion exercises with BFR applied to the proximal limbs until task failure with 30–60 seconds rest between sets. The pressure cuff will be placed on thigh, close as the groin area, when performing leg extension and plantar flexion exercises, and upper arm, just below the shoulder joint, when performing elbow flexion exercises. The cuff pressure for each limb will be regulated by KAASTU Master (KAATSU Training Japan Co., Ltd., Tokyo, Japan) and determined on each day of exercise for an individual. The cuff pressure will be initially applied and released in increments of 20 mmHg. This pressure on-pressure off sequence will continue until the circulation in the limbs is impeded, but not occluded. Specifically, the cuff pressure will be set when the capillary refill time of the leg just above the knee (for the leg pressure cuff) or the palms of the hands (for the arm pressure cuff) is between 2 and 3 seconds. The pressure cuffs will remain inflated until the completion of all three sets of exercise, including the rest periods. The inflated pressure will be increased progressively based on the capillary refill time and subject tolerance throughout the 10-week period. After completing the BFR exercises, subjects in the BFR exercise group will perform 3 sets of 15 repetitions of trunk extension exercises at 25 % of maximal isometric strength. During all exercises study participants will be reminded to contract their abdominal muscles by retracting their umbilicus.

The control exercise group will perform an identical exercise protocol as the BFR exercise group except that BFR will not be applied to the appendicular limbs. If subjects in either group are able to perform 70 repetitions in a given set prior to reaching task failure, they will be asked to stop the respective set. An overview of the study groups is illustrated in Table 3.

**Table 3:** Overview of the study groups

Group	Blood flow restriction	Frequency	Exercise regimen
Exercise control group	No	Twice weekly	• 3 sets of leg extension, calf raises, and arm curls at 25 % of individuals’ isometric MVC to failure (30–60 seconds rest between sets) • 3 sets of trunk extension at 25 % for 15 repetitions (30–60 seconds rest between sets) • Up to 3 minutes rest between each exercise station
BFR exercise group	• Pressure cuffs applied to the upper leg during legs exercises and upper arms during arm curls • Pressure is maintained throughout the 3 sets for the respective exercises	Twice weekly	• 3 sets of leg extension, calf raises, and arm curls at 25 % of individuals’ isometric MVC with BFR to failure (30–60 seconds rest between sets) • 3 sets of trunk extension at 25 % of individuals’ isometric MVC for 15 repetitions (30–60 seconds rest between sets) with BFR on upper arm • Up to 3 minutes rest between each exercise station

*BFR* blood flow restriction, *MVC* maximum voluntary contraction

### Sample size calculation

We assume an attrition rate of 10 % for the muscle CSA and muscle function outcomes immediately following training and 20 % for the pain and disability outcomes at follow-up, both assuming missing at random. We also assume that three covariates (e.g., pre-training value, sex, age, etc.) will explain an additional 30 % of the unexplained variance for all outcomes (correlation between the baseline volume and the percentage change in our pilot data was *r* = −0.57). Significance will be set to 0.05, and no *p* value adjustment will be applied due to the exploratory nature of the study. For the primary outcome, our pilot data indicate that the control exercise group and the BFR exercise group differ in CSA percentage change by 3.1 ± 3.1 %. The standard deviation and adjusted effect size derived from our small sample of pilot data (*n* = 7) are, as expected, imprecise: 95 % confidence interval (CI95) around the standard deviation (SD) = 2.0–6.9; around Cohen’s *d* = −1.1–3.2. As such, the primary goal of this study is to gather critical preliminary data to accurately and precisely estimate the population effect size so that future studies will be adequately powered. We will achieve accurate estimation by minimizing potential bias through designing a better study: blinding, randomization, and examining missing patterns. We will achieve more precise estimation by more than doubling the sample size to at least *n* = 16/group (and will enroll up to 20/group depending on resources). This sample size will narrow the range of CI95 around the estimated population effect size by over 60 %, from 3.2–(−1.1) = 4.3 to 1.9–0.3 = 1.6. This sample size will also allow us to achieve a desired power for the future study more than 95 % of the time by overestimating the population SD [70]. For reference and planning purposes we have conducted a power analysis. To make our test conservative, we estimated a treatment effect of 2.5 %. If we assume its SD to be in the range of 3.0–6.0, which will be translated into adjusted Cohen’s *d* of 0.42 to approximately 0.83 the corresponding sample size to achieve a power of 0.8 would be 26–100 per group, taking attrition into consideration. Likewise, required sample sizes would be 30–65 per group for trunk strength and endurance, assuming 6.0 ± 8.0–12.0 % improvements, and 41–112 per group in pain and disability averaged over the course of a year assuming 2.0 ± 3.0–5.0 and 4.0 ± 6.0–10.0 reductions in pain and disability, respectively.

### Statistical analyses

For the three trunk extensor-related outcomes (i.e., size, strength, and endurance), we will compute a percentage change and will test a difference in group means using linear mixed-effects (LME) models with covariates included to increase the precision of effect-size estimation. To analyze LBP recurrence, we will compute the rate of LBP recurrence, average duration of a LBP episode, and intensity as the mean and maximum pain and disability scores over the follow-up period as well as during an episode of LBP occurrence. We will test whether the groups differ in these measures with LME models. We will assess potential bias due to loss to follow-up by examining whether characteristics measured at baseline and immediately following the training will predict attrition. We will perform exploratory intention-to-treat analyses (ITT), of all randomized study participants who have baseline assessments and estimate parameters based on last observation carried forward as well as maximum likelihood estimation. We will also perform exploratory per-protocol analyses (PPA) where we will exclude study participants who (1) fail to attend 75 % of their exercise training sessions, (2) receive prohibited concomitant interventions, or (3) develop an exclusionary medical condition while on study protocol.

## Discussion

This is the first randomized controlled trial investigating the potential for BFR exercise applied to appendicular muscles to result in a “cross-transfer” of therapeutic effect to the lumbar musculature in individuals with LBP. Madarame et al. reported that 10 weeks of BFR exercise facilitated a “cross-transfer” effect to other skeletal muscles, with the “cross-transfer” effect resulting in an 11 % increase in muscle size. Our pilot data, which were obtained from individuals without LBP, but who are “at risk” for the development of recurrent LBP based on exhibiting poor trunk muscle endurance [21, 23], indicated that BFR exercise resulted in an approximate 4 % increase in TE CSA (unpublished data). Effectively, we anticipate that persons with recurrent LBP and poor muscle endurance will exhibit an even greater enhancement in muscle size associated with BFR exercise.

The amount of TE hypertrophy needed to exert a clinically meaningful change is not known; however, our expected outcomes would be sufficient to nearly, or completely, reverse the amount of atrophy (6–7 %) observed in persons with recurrent LBP, where atrophied muscles have been associated with the frequency of LBP [71]. While the expected amount of TE hypertrophy may seem small, it should be noted that the anticipated results would be impressive, as even extremely aggressive interventions designed to increase muscle size often produce substantially less hypertrophy (e.g., averaged treatment effect for testosterone therapy lasting 11 months is 2.7 %) [72]. With regards to strength, our pilot data showed that BFR exercise increased TE strength by 13 %. While clinical meaningfulness of the changes in strength and LBP outcomes has not been established or examined, the increase in strength observed in our pilot data would be considered to exert a minimally important difference on physical function in other conditions/populations (e.g., older adults) [73].

The largest potential problem that could arise in the course of this study relates to the possibility for adverse events (AE). BFR exercise is popular in Japan (known as KAATSU training), and surveys of Japanese facilities (at least 30,000 BFR exercise sessions) indicate the most common side effects to be subcutaneous bruising at the cuff location (13.1 %), numbness (1.3 %), and lightheadedness (0.3 %) [74]. We, and others, have also reported that BFR exercise does not alter prothrombin time or markers of coagulation [48, 57, 75], nor does it alter arterial stiffness or nerve conduction [48]. Thus, we do not anticipate significant issues related to AE. Throughout the study and follow-up period we will monitor safety and closely report all AE.

This study will provide important insight into the effectiveness of BFR exercise in recurrent LBP. If it is found to be effective to treat recurrent LBP, this novel exercise modality will provide the foundation for a cost-effective and easy-to-implement rehabilitation strategy that is superior to existing paradigms in its capacity to induce muscle adaptation in the absence of high mechanical and compressive loading on the spine, which could be detrimental for individuals with recurrent LBP.

## Trial status

Actively recruiting. Start date: January 2015. Expected completion date: January 2017.

## Abbreviations

AE: adverse events
BEND: Back Exercises to Neutralize Disability
BFR: blood flow restricted/restriction
CI95: 95 % confidence interval
CSA: cross-sectional area
DEXA: dual-energy X-ray absorptiometry
ITT: intention-to-treat analyses
LBP: low back pain
LME: linear mixed-effects
MRI: magnetic resonance imaging
PPA: per-protocol analyses
SD: standard deviation
TE: trunk extensor
